# Researching children's individual empathic abilities in the context of their daily lives: the importance of mixed methods

**DOI:** 10.3389/fnins.2015.00261

**Published:** 2015-07-28

**Authors:** Simone Roerig, Floryt van Wesel, Sandra J. T. M. Evers, Lydia Krabbendam

**Affiliations:** ^1^Department of Educational Neuroscience, Faculty of Psychology and Education, VU University AmsterdamAmsterdam, Netherlands; ^2^Department of Methodology and Statistics, Faculty of Social and Behavioural Sciences, Utrecht UniversityUtrecht, Netherlands; ^3^Department of Social and Cultural Anthropology, Faculty of Social Sciences, VU University AmsterdamAmsterdam, Netherlands

**Keywords:** mixed methods, empathy, social neuroscience, anthropology, participant observation, social network analysis, children

## Abstract

In social neuroscience, empathy is often approached as an individual *ability*, whereas researchers in anthropology focus on empathy as a dialectic *process* between agents. In this perspective paper, we argue that to further elucidate the mechanisms underlying the development of empathy, social neuroscience research should draw on insights and methods from anthropology. First, we discuss neuropsychological studies that investigate empathy in inter-relational contexts. Second, we highlight differences between the social neuroscience and anthropological conceptualizations of empathy. Third, we introduce a new study design based on a mixed method approach, and present initial results from one classroom that was part of a larger study and included 28 children (*m* = 13, *f* = 15). Participants (aged 9–11) were administered behavioral tasks and a social network questionnaire; in addition an observational study was also conducted over a period of 3 months. Initial results showed how children's expressions of their empathic abilities were influenced by situational cues in classroom processes. This effect was further explained by children's positions within classroom networks. Our results emphasize the value of interdisciplinary research in the study of empathy.

## Introduction

“You do an empirical experiment and you get an empirical result. What can any anthropologist tell me that could change that?” (comment of a neuroscientist in Whitehead, 2012, p. 44)

The human brain is exquisitely designed to navigate the social world and its complexity of social networks. The mechanisms underlying this ability have been elucidated by a large body of social neuroscience research. This has led to the delineation of a brain network, termed the “social brain,” that implements our ability to understand others and interact with them (Lieberman, 2007; Adolphs, 2009; Frith and Frith, 2010). It is noteworthy that progress in social neuroscience has been largely independent from advances in social science research, yet the interplay between social networks and social cognition is at the core of human social functioning. This is particularly the case during childhood and adolescence, as social development is clearly driven by maturation of specific social cognitive functions, yet these functions do not develop in isolation but are shaped by interpersonal relationships within social networks (Crone and Dahl, 2012). In this perspective paper, we argue that to further understand the mechanisms underlying development of social cognition, social neuroscience research should draw from insights and methods of the social sciences, and particularly those of anthropology. Based upon an overview of studies that investigate social cognition in the context of interpersonal relationships, focusing on empathy, we pinpoint the key differences between the social neuroscience and anthropological conceptualizations of empathy. We then introduce a new study design and present initial results to demonstrate how methods drawn from anthropology can be combined with neuropsychological approaches to shed further light on the interplay between social cognition and social context.

## Social cognition in context

Two crucial processes underlying our ability to understand others are (i) cognitive empathy, or the ability to take another person's perspective into account and attribute intentions, desires, and beliefs; and (ii) affective empathy, or the ability to share other's feelings by observing or learning about their emotional state (Decety and Lamm, 2006). Cognitive empathy is often assessed using tasks that require inferring the mental state of a protagonist, such as false belief tasks. The behavioral assessment of affective empathy often relies on self-reported intensity and valence of vicarious emotions in hypothetical situations, while neuroimaging research focuses on the actual vicarious response toward emotional stimuli. The cognitive aspects of empathy rely upon a network including medial prefrontal cortex, temporoparietal junction and precuneus; and affective empathy is based on activation in similar brain regions as experiencing the pain or emotion oneself, i.e., anterior insula, anterior cingulate cortex, amygdala (Decety and Jackson, 2004; Lieberman, 2007; Frith and Frith, 2010).

The first set of studies investigating empathy in a social context focuses on the relationship between empathy and the nature and number of interpersonal relationships. For example, the size of social network was positively associated with cognitive empathy and this effect mediated the association with the volume of the ventromedial prefrontal cortex (Lewis et al., 2011; Powell et al., 2012). Associations have also been reported between size and complexity of social networks and other structural brain characteristics, such as amygdala gray matter density (Kanai et al., 2012) and volume (Bickart et al., 2011), that are thought to be involved in empathy and other social cognitive functions. These studies build on a larger body of behavioral research, suggesting that cognitive and affective empathy are positively associated with characteristics of one's social environment, such as the presence of older siblings (Perner et al., 1994; Wright and Mahfoud, 2012), and a central position in one's social network of friends (Wölfer et al., 2012).

The second set of studies focuses on how the expression of empathy is affected by specific characteristics of the current social context, specifically the characteristics of the target eliciting the empathic response. Distance between the target and the empathizer has been shown to dampen the empathic response, whether distance is created by social, racial, political, spatial, or temporal features (Cikara et al., 2011). These behavioral findings were also confirmed in neuroimaging studies. That is, empathy for friends relied more strongly upon mechanisms associated with affective sharing and self-processing (dorsal anterior cingulate and medial prefrontal cortex); whereas empathy for strangers elicited activation involved in thinking about others (dorsal medial prefrontal cortex, precuneus, anterior temporal cortex, Meyer et al., 2013). These findings extend to interacting with similar vs. dissimilar others (Mitchell et al., 2006; Mobbs et al., 2009; Xu et al., 2009; Eres and Molenberghs, 2013).

## Anthropology and empathy

This short discussion of illustrative studies shows that interpersonal relationships are taken into account in the study of social brain and cognition. However, even in such research, empathy is fundamentally considered to be a characteristic of the individual. By contrast, in anthropology empathy is seen as “an ongoing, dialogical, inter-subjective accomplishment that depends very much on what others are willing or able to let us understand about them” (Hollan and Throop, 2008, p. 394). Most anthropological research is therefore focused on understanding how social and cultural processes may inhibit or enable the expression of empathy in a given situation with particular people (Hollan and Throop, 2011). In these dynamics, the target becomes just as important as the empathizer (see Groark, 2008; Hollan, 2012).

Two clear examples of the importance of these dynamics appear in the work of Briggs (2008) and Hollan and Throop (2011). The former shows how among the Inuit expressing one's emotions publicly is seen as something childish, resulting in the fact that being “empathized” is something adults would rather avoid than support. Hollan and Throop (2011) then describe how in the Yap society (western Pacific Ocean) the empathy dynamics between empathizer and empathized are directly related to social status. Being compassionate places one in the higher position which makes getting involved in this process a tricky enterprise for both people with lower and higher status. In both cases, emotions become part of a hidden social dynamic as keeping one's mental state to oneself is socially valued. This is also recognized by other anthropologists who define this as “opacity of mind”/“social opacity” (Groark, 2008; Astuti, 2015). This means that cultural factors should be assessed as a component of the social interaction: people act as part of a wider social network and expressing empathy might be consciously (through individual agency) or unconsciously concealed.

It is also important to assess how individual's learned behavior on empathic skills (“how empathy has to be performed”) might deviate from how they actually behave (“how empathy actually is performed”). This can be usefully assessed through participant observation as methodologically refined by anthropologists (see Astuti, 2015).

## Ability or process: Different conceptualizations of empathy and its consequences

The social neuroscience focus on individual abilities and individual differences contrasts with the anthropological focus on understanding empathic processes as embedded in social relations. Taking this distinction one step further, it can be argued that social neuroscience focuses predominantly on internal mechanisms, whereas anthropology emphasizes external (principally expressed) human thoughts and behaviors. For both perspectives, it is important to be clear about which particular facet of social cognition is being studied (Astuti, 2015).

Obviously, there are also methodological implications related to the different foci in the two fields. In anthropology, social dynamics in everyday life are often captured by the method of participant observation. An anthropologist thus becomes part of the daily lives of the participants by effectively participating in a maximum of aspects of their social life, while systematically taking observational notes on the subject one wishes to understand (among others Herbert, 2000; Astuti, 2015). Often these observational field notes are accompanied by reflections of the researcher on one's subjectivity and qualitative interviews (see for example, Wolfinger, 2002). Subsequently, this qualitative data is systematically analyzed, resulting in a written narrative of the findings (see for example, Corbin and Strauss, 2008). In contrast, in neuroscientific experiments, the subjectivity of the researcher is removed insofar as possible and separable components of social cognition are isolated and in- or excluded.

Our main point here is that to capture a rich concept such as human empathy we believe social neuroscience can gain by taking anthropological perspectives into account; and vice versa. This idea is supported by Hollan (2012) and Keysers and Gazzola (2014) who call for scholars to research expressed empathy in relation to empathic ability, and combine situational ethnographic data on empathic processes with individual ability data on people's tendency to empathize. Astuti (2015) implies the same integration when she advocates how the anthropological understanding from within, i.e., actually participating in people's lives to understand their norms, values and daily life, can form the proper contextualization underlying specific experimental tasks. Some scholars have already taken up this challenge. For example, Astuti (2007), weaved culture and cognition into the analytical component of her research project in Madagascar. Roepstorff and colleagues then, proposed the patterned practices approach to explain and detect the interaction between neural activity and social context (Roepstorff et al., 2010), and highlighted the concept of agency to better understand what is actually going on in experiments in social neuroscience (Roepstorff and Frith, 2004). However, conceptually and empirically, a gap remains between the social neuroscience focus on individual abilities assessed in laboratory experiments and the anthropological focus on the dynamics of social relationships assessed by interviews and participant observation.

In the following section, we therefore introduce a research design and initial results which aim to integrate these two foci in an interdisciplinary study. This entailed a mixed method approach (see Johnson et al., 2007), using behavioral tasks to investigate empathic ability, participant observation to investigate the actual expression of empathy in daily life, and social network analysis to understand how social relationships impact on this expression. As this integrative approach is somewhat novel, we elected to restrict our focus to behavioral empathy tasks, with a view to subsequently providing a platform for neuroimaging studies.

## Study background

The aim of the study was to investigate empathy in children, (i) measured as an individual ability in optimal experimental settings, divested insofar as possible of the influence of social context; and (ii) as expressed in their everyday (school-)lives, focusing on children's agency and their social relationships in the classroom, including both peer relations and children's relations to adults, such as teachers, parents etc. The research is focused on children aged 9–11. Since empathy is still developing, assessing the interplay with social context is even more important. At the same time, from the age of 9 empathic abilities such as understanding the mixed nature of emotions, the possibility of regulating emotions via cognition and the influence of morality on emotions are present (Pons et al., 2004). Finally, a primary school with school classes offers a suitable context for conducting social network analysis.

## Methods

### Participants and procedure

Four schools in the Netherlands participated in the study. The schools were selected from the professional network of the researchers and both the children and their parents signed the informed consent form before participating. Moreover the study was approved by the Ethical Committee of the Department of Psychology and Education of the VU University. The current analysis focused on one classroom from one of these schools. The class was comprised of 28 children (13 boys, 15 girls) in grade 4, aged 9–11 years old. The school population was mainly native Dutch. The behavioral tasks and social network questionnaire were administered in random order. Children went outside the classroom and completed their tasks individually and anonymously. All tests were administered in the presence of one of the researchers. To assess cognitive empathy, we administered three tasks that were designed to measure the ability of understanding the mental state of other people: Reading the Mind in the Eyes test for children (Baron-Cohen et al., 2001), the Level of Emotional Awareness Scale for children (LEAS-C) (Bajgar et al., 2005) and the self-report: How I Feel in Different Situations (Caravita et al., 2009) which consisted of five cognitive and six affective items. The focus of the current paper is on the LEAS-C. The LEAS-C measures the ability to understand the complexity of emotions, distinguishing between feelings of self and others. The LEAS-C/Other score reflects the way in which children report the feeling of the other person in a specific simulated situation; this could be the mother, a classmate, a friend, whereas the LEAS-C/Self score stands for the way in which children report their own feelings in this same fictive situation. The social network questionnaire consisted of questions about their current relationships. We asked the children to report for each of their classmates either a like, a dislike, or a neutral relation. Here we report the number of like and dislike elections made by a participant (outdegree) and the number of like and dislike nominations a participant receives (indegree) (see for these terms Wasserman and Faust, 1994). For the participant observation, the researcher was present in the classroom one school day per week for a period of 3 months, participated in the daily routine of the group (Herbert, 2000).

### Data analysis

The social network analysis was conducted using UCINET (Borgatti et al., 2002), focusing on the indegree and outdegree ties in the like and dislike networks. In UCINET and the related program NETDRAW (Borgatti, 2002) first the “like”/“dislike” network of the classroom based on indegree and outdegree was visualized, and thereafter the scores of an ability test—in this case LEAS-C—was attributed to each child. Subsequently, ability scores were linked to the children's position in the classroom network. The qualitative data, especially the field notes, were analyzed using NVivo10 (QSR International Pty Ltd. Version 10, 2012), a software for qualitative data analysis (see Richards, 1999). A thematic analysis was conducted consisting of open, axial and selective coding (Corbin and Strauss, 2008; Boeije, 2010). At every stage in the analysis described below, findings were discussed and agreed upon by at least two researchers. These results are not reported in terms of quantities, but in written narratives based on thorough analysis. Finally, individual ability scores were analyzed in relation to observational data, investigating to what extent that ability is expressed in daily life and both corroborative and discordant findings were further tested using the results from the social network analysis.

## Results and discussion

Mean score on the LEAS-C total (possible scores 0–60) was 34.4, *SD* = 5.5; range 21–50; mean score on the LEAS-C self (possible scores 0–60) was 31.8, *SD* = 6.5; range 19–49; mean score on the LEAS-C other (possible scores 0–60) was 29.2, *SD* = 7.3; range 8–36. The like-ties in the social network yielded the following scores: outdegree mean score = 7.2; *SD* = 2.4; range 3–12 and the indegree mean score = 7.2; *SD* = 3.2, range = 1–14. Dislike scores were as follows: outdegree mean score = 6.8; *SD* = 4.2; range 0–14 and the indegree scores: mean = 6.8; *SD* = 3.0; range 0–13.

Based on the analysis of the observational data, two patterns in expressive empathy were detected. First, the presence of the teacher, or in some cases another adult, stimulated the children to verbalize and activate their empathic abilities. For example, when the teacher initiated a discussion on “the rule of the week: be a good classmate” in the classroom, the children were eager to define with the teacher which behavior was desirable and which was not. However, 1 h later during morning break six girls were excluded from the game by the other girls, resulting in a different application of “being a good classmate” from the model recently adopted inside the class. This change of behavior in the presence of an adult might not be surprising, but does highlight how socio-cultural context conditions the willingness to express empathy, providing an example of the importance of individual choice or agency. The second pattern highlighted the importance of “who is *in,”* meaning: who belongs to my group at this particular moment. For example, a weeping classmate triggers different empathic behavior than a crying friend. The weeping classmate can at times be ignored or put aside “he/she is always crying,” whereas the crying friend is more likely to be comforted. This highlights the importance of the dynamics between empathizer and empathized, which will be further elucidated in the analysis of children's social networks.

Examining the relationship between the scores on the LEAS-C/Other and the observational data suggested that similar (high) ability levels—the individual scores obtained in the behavioral tasks that assess the individual tendency to be empathic—may be expressed differently by different children. Specifically, three main types of behavior were observed: the first was characterized by friendliness toward close friends, but provocation toward others; the second was defined by getting along with everyone in an overtly friendly matter but minimal involvement in emotionally charged situations; the third was marked by showing consideration toward most children, combined with a selective choice of friends. Correlating the observational findings with the social network analysis helped to shed light on these apparent discrepancies: the first type of behavior could be observed in children who had many ties in a specific female part of the network, but also a relatively high indegree of dislikes from both boys and girls; the second had incoming ties from the whole network, resulting in a high indegree of likes and scored close to zero indegree *and* outdegree of dislikes; and the third type had a high indegree of likes among the female group combined with a fairly low indegree of dislikes. Thus, as described above, children with similar high LEAS-C/Other scores all used their empathic abilities actively, but in varying manners. Moreover, these differences could be understood as a function of network position. There were also children where our social network findings did directly match expectations based on ability scores and observational information.

In sum though, whether or not children's abilities at the level of knowing were transformed into their actual expression of empathy in classroom processes was influenced by children's situational motivations resulting from the presence or absence of certain actors at a particular moment. This process could be explained by children's position in the network, especially for children with relatively high ability scores.

## Concluding remarks

In this perspective paper we argue for the importance of bridging neuropsychology and anthropology in the research of children's empathy. We proposed a research design that incorporated the examination of both empathy as an individual ability and empathy as a socio-culturally embedded process. Preliminary results suggest that the contextual information which was collected through participant observation and social network analysis is crucial to understand the ways in which individual children make use of their cognitive abilities. Both the dialectic processes between children in and outside the classroom and children's individual abilities were relevant to gaining better insights in children's empathy. Without information on children's daily interactions the differential expressions of empathy by children with similar ability scores might have escaped detection. Likewise, based on the observational data only, a lack of participation in the social processes in the class would be difficult to interpret unambiguously, as it may be due to a lack of ability, but equally to a specific position in the network. We therefore argue, as does Hollan (2012), in favor of designing studies that scrutinize the relationship between empathic ability and empathic expression, thus taking into account both the tendency to empathize and the socio-cultural contexts within which empathic behaviors occur. We conclude this paper with a response to the rhetorical question put by the neuroscientist cited at the outset of this article: “You do an empirical experiment and you get an empirical result. What can any anthropologist tell me that could change that?” The short answer to this query is that the purpose of the exercise is to add to the conceptual toolbox of scientists researching children's empathic capabilities. An interdisciplinary approach need not be achieved at the expense of mono-disciplinary scientific strength (see also Roepstorff and Frith, 2012, p. 105). The combining of a neuroscientific approach with anthropology is nothing more, nothing less than positing the study of social cognition within its broader socio-culturally embedded context. Our role is not to change, but to sharpen the scientific lens through which we view children's empathy.

### Conflict of interest statement

The authors declare that the research was conducted in the absence of any commercial or financial relationships that could be construed as a potential conflict of interest.
